# Direct Detection of Methicillin-Resistant Staphylococcus Aureus in Sputum Specimens from Patients with Hospital-Associated Pneumonia Using a Novel Multilocus Pcr Assay

**DOI:** 10.3390/pathogens4020199

**Published:** 2015-04-30

**Authors:** Zeng-Guang Huang, Xing-Zheng Zheng, Jing Guan, Shu-Nian Xiao, Chao Zhuo

**Affiliations:** 1 State Key Laboratory of Respiratory Diseases, the first affiliated hospital of Guangzhou Medical University, Guangzhou, 510120, China; E-Mails: dyshxdtkml@163.com (Z.G.H.); g-jing.27@163.com (J.G.); 240671094@qq.com (S.N.X.); 2 Peking University Health Science Center, Beijing, 100191, China; E-Mail: zxz806@bjmu.edu.cn

**Keywords:** MRSA, *mecA* gene, *nuc* gene

## Abstract

Methicillin-resistant *Staphylococcus aureus* (MRSA) is a significant cause of hospital-associated pneumonia (HAP). The rapid identification of MRSA would be beneficial for early diagnosis. The study aimed to evaluate a multilocus, fluorescence-based PCR assay based on the detection of *mecA* and *nuc* genes for identification of *S. aureus*in lower respiratory tract (LRT) specimens. Sensitivity and specificity of the PCR assay were analyzed. Clinical evaluation for the assay was performed using LRT specimens from patients with HAP, and the sensitivity, specificity, positive and negative predictive values (PPV and NPV) were evaluated in comparison with semi-quantitative culture methods. The result showed the assay provided positive identification of all MRSA reference strains with a limit of detection for MRSA of 4 × 10^3^ CFU/mL. Compared with semi-quantitative culture, the sensitivity, specificity, PPV and NPV were 100%, 89.6%, 75.0%, and 100%, respectively. A positive correlation between MRSA bacterial colonies and PCR copy number was found. The specificity and PPV reached 96.6% and 89.7% respectively, if the PCR copy number reached a definite positive threshold of 5.96 × 10^5^. It suggested that this novel multilocus, fluorescence-based PCR assay proved to be a fast, sensitive and specific tool for direct detection of MRSA from LRT specimens.

## 1. Introduction

Staphylococci bacteria, including MRSA, are some of the most common causes of hospital-associated pneumonia (HAP). Prospective surveillance programs at 73 hospitals in 10 Asian countries between 2008 and 2009 revealed that *S. aureus* was the isolate most frequently identified as a definite or probable cause of HAP and ventilator-associated pneumonia (VAP) (15.6%). Furthermore, 82% of *S. aureus* isolates were identified as MRSA [1]. Similarly, a prospective, multicenter study in China between August 2008 and December 2010 showed that *S. aureus* was the third most frequent isolate (15.6%) identified in 610 cases of HAP, and MRSA accounted for 87.8% of *S. aureus* isolates. *S. aureus* was also a frequently identified pathogen (45.7%) in cases with a score of more than 20 based on the acute physiology and chronic health evaluation II (APACHE II) as compared to cases caused by other pathogens [2]. The associated mortality for patients with MRSA infection is twice as high as for patients with methicillin-susceptible *S. aureus* (MSSA) infection [3]. The costs of managing patients with MRSA infection are 1.5–3-fold higher than for patients with MSSA infection [3,4]. 

In order to effectively eliminate MRSA infections, early diagnosis and species identification are of paramount importance. However, conventional culture-based systems take one to two days to grow and an additional one to two days for identification of the MRSA phenotype, which often leads to considerable delays in initiation of targeted treatment [5]. In 2002, ChromID MRSA (bioMérieux, Marcy l’Etoile, France), one kind of chromogenic agar medium, was introduced to laboratory, which resulted in a reduction of reporting time of around one day rather than days incubation. Furthermore, several DNA-based tests have been developed for the rapid detection of MRSA including GeneXpert MRSA and BD GeneOhm MRSA, which are based on single locus PCR detection of the *mecA* gene, and the LightCycler Staphylococcus + MRSA Kit, which is based on multilocus PCR detection of the target region including the right-extremity attachment site for SCC*mec*, the left-extremity attachment site for SCC*mec*, the intact *orfX* (without SCC*mec*), the *mecA* gene, and the internal transcribed spacer (ITS) region to generate amplicons for sequencing [6,7,8]. Many methods for detection of MRSA, however, are limited to the use of *S. aureus* cultures or sterile sample such as blood and less bacteria samples such as nasal swabs, and nasal aspirations, which should not contain other organisms. In fact, clinical respiratory tract samples often contain both coagulase-negative staphylococci (CoNS) and *S. aureus*, either of which can carry *mecA*, even in healthy population [9]. In a mixed flora clinical sample, MRSA and methicillin-resistant CoNS cannot be sufficiently discriminated by detection of the *mecA* gene [10]. When these tests are used for direct detection of MRSA in clinical specimens containing both, the results may be non-specific, resulting in a higher false-positive rate [6]. Specimens from the lower respiratory tract (LRT), including sputum, endotracheal aspirate (ETA) and bronchoalveolar lavage fluid (BALF), have been the mainstay for diagnosis of HAP. In the ANSORP survey, blood cultures accounted for 11% of specimens and samples from the LRT accounted for 60% of the microbiological specimens from patients for diagnosis of HAP [1]. Diagnostic kits for MRSA in LRT specimens may, therefore, be of significant use for the early diagnosis of MRSA-associated HAP.

*nuc* is an extracellular nuclease, and also an *S. aureus*-specific gene[11]. This gene is always being used as a molecular target for the identification of *S. aureus* [12]. Therefore, one approach to address specificity issues with PCR-based assays for detection of MRSA involved the development of a multilocus, fluorescence-based PCR protocol for detection of the *mecA* and *nuc* gene combination (Triplex International Bioscience Co. Ltd, Fujian, China), which was found to be suitable for routine use in a variety of clinical specimens, including blood, nasal swabs, sputum and BALF. Its quantitative analysis also assists in identification of MRSA colonization from sputum samples originating from HAP patients. Here, we report on the development of a multilocus, fluorescence-based PCR assay for direct detection of MRSA and MSSA in clinical sputum specimens from HAP patients. The sensitivity, specificity, PPV and NPV were evaluated in comparison with the standard culture method. 

## 2. Experimental Section 

### 2.1 Study Population, Sample Collection, and Bacterial Culture

We performed a prospective study between June 2012 and June 2013. A total of 361 sputum specimens, including purulent sputum coughed up by patients and sputum from ETA, were collected from patients with HAP at a teaching hospital in Guangzhou, China. All samples examined in the study were confirmed to meet the microbiological standard of more than 25 squamous leukocytes and less than 10 squamous epithelial cells per field at low magnification. Each sample was divided into two parts for parallel analysis, one that was analyzed by standard culture methods, and another analyzed by PCR. 

HAP was diagnosed based on ATS/IDSA guidelines [13], which identify pneumonia based on infiltration on chest x-ray and clinical findings (body temperature of more than 38.5 °C or less than 36 °C, leukocytosis or leukopenia). Patients who developed a new pneumonia infection on mechanical ventilation for more than 96 h were diagnosed as having ventilator associated pneumonia (VAP) [13].

Sputum samples were processed with the addition of an equal volume of Sputasol (OXOID, Hampshire, UK) and several glasses beads (1–1.5 mm in diameter) and incubated for 30 min at 37 °C, followed by intermittent vortexing for 5–10 s. A ten-fold serial dilution of the homogenized sample was carried out in brain-heart infusion broth and 100 µL aliquots were spread on blood agar. Plates were incubated for 18 to 24 h at 35 ± 2 °C aerobically, and bacterial colonies were counted and subcultured for identification by biochemical analysis following the manufacture’s protocol (API 20-NE System; BioMérieux, Marcy-L’Etoile, France) or analyzed on an automated system (Vitek; BioMérieux). Colony counts of ≥10^7^ CFU/mL for coughed sputum, or ≥10^4^ CFU/mL for ETA of either MRSA or MSSA indicated pathogenic HAP (quantitative culture), while the remaining cultures were considered as colonization [14]. The *S. aureus* strains ATCC 25923 and ATCC 33592 were used as references for MSSA and MRSA, respectively.

### 2.2 Standardization of Real-Time PCR

Following addition of 0.1 N NaOH, DNA was extracted from sputum samples through a series of steps including high speed centrifugation, using the *S. aureus* and MRSA Nucleic Acid Detection Kit (Fluorescent PCR, SFDAN3400251), supplied by Triplex International Bioscience (China) Co. Ltd (Fujian, China). Specific primer sequences and probes were designed to detect *nuc* and *mecA*. The *nuc* and *mecA* probes were tagged with FAM and HEX fluorescein, respectively. The *neo* gene was using as an internal control for real-time PCR [15]. To avoid contamination of PCR products, uracil-N-glycosidase and dUTP were added. In addition, the kit included DNA extraction reagents, positive and negative controls. The PCR cycling profile included 4 stages: (1) 37 °C for 2 min; (2) 94 °C for 2 min; (3) 94 °C for 20 s and 55 °C for 45 s, 10 cycles; (4) 94 °C for 20 s and 55 °C for 45 s, 25 cycles. The fluorescence signal was collected at this stage. The presence of the *nuc* gene identified the presence of *S. aureus*, and the presence of *mecA* in *S. aureus* strains indicated the presence of MRSA.

### 2.3 Analytical Sensitivity and Specificity of the Multilocus Fluorescence-Based PCR Assay 

Evaluation of analytical sensitivity and specificity of the assay defined according to Holfelder’s reports [5]. Briefly, the sensitivity of the assay was evaluated using 10 MRSA clinical isolates, 10 MSSA clinical isolates, and the reference strains ATCC 25923 (MSSA) and ATCC 33592 (MRSA). The 10 MRSA clinical isolates were pre-confirmed to represent SCC*mec* types I, II, III, IVa and IVb. All strains were incubated for 24 h on Columbia blood agar plates, and cultures adjusted to the McFarland 0.5 standard suspension (1 × 10^8^ CFU/mL). The suspension was then serially diluted ten-fold in lysis buffer with sterile NaCl to a final concentration of 10 CFU/mL. DNA from each dilution was extracted according to method described for the multilocus, fluorescence-based PCR assay. A positive quantitative PCR signal for the lowest concentration of the suspension was defined as the lower limit of detection for the assay.

The analytical specificity of the assay was determined using 30 methicillin-resistant CoNS and methicillin-susceptible CoNS samples from our laboratory, including five samples of methicillin-resistant *S. epidermidis*, five samples of methicillin-susceptible *S. epidermidis*, five samples of *S. haemolyticus*, and one each of *S. hominis*, *S. lugdunesis*, *S. capitis*, *S. auricularis*, *S. caprae*, *S. carnosus*, *S. cohnii*, *S. delphini*, *S. equorum*, *S. gallinarum*, *S. intermedius*, *S. sciurii*, *S. xylosus*, *S. warneri* and *S. schleiferi*. In addition, seven streptococcus strains, reported to be common in colonization or infection of the throat and respiratory tract were analyzed, including two samples of *S. mitis*, two of *S. parasanguinis*, two of *S. anginosus*, two of *S. salivarius*, two of *S. pneumoniae*, two of *S. adjacens* and one sample of *S. constellatus* subsp. pharyngis. 

### 2.4 Statistical Analysis 

Specificity was defined as the number of negative results obtained by real-time PCR divided by the number of negative results by the quantitative culture test. The sensitivity was determined as the number of positive results by real-time PCR divided by the number of positive results by quantitative culturing. Positive predictive value (PPV) and negative predictive value (NPV) were also calculated.

Early diagnosis of this PCR assay was assessed. The specimens randomly selected from HAP patients were subjected to be collected every other day in two weeks of diagnosis, resulting in seven specimens from each patient. Each sample was divided into two parts, one was analyzed by standard culture method, and another analyzed by PCR. With the comparison to standard culture method, early diagnosis of this PCR assay was assessed through the time of positive results appearing.

To compare the number of MRSA or MSSA samples as determined by real-time PCR to those determined by standard culture methods, the nonparametric procedure sign test (SPSS software package, version 11.0) and a two-by-two matrix were used. 

## 3. Results

### 3.1 Analytical Sensitivity and Specificity of the Multilocus Fluorescence-Based PCR Assay

All 10 clinical MRSA isolates and the reference strain ATCC 33592 tested positive based on *mecA* and *nuc* gene identification by multilocus fluorescence-based PCR, while 10 clinical MSSA isolates and the reference strain 25923 tested positive with identification of only the *nuc* gene. The lower limit of detection for the assay using serially diluted MRSA strains showed a detection limit of 4 × 10^3^ CFU/mL.

All 30 CoNS tested, including *mecA* positive CoNS, as well as five MSSA strains, were identified as negative. The assay also revealed negative results for the seven streptococcus strains. Compared to standard bacterial culture methods, the assay had a sensitivity of 100% and an analytical specificity of 100%.

### 3.2 Clinical Sample Detection

A total of 154 samples were collected from 135 HAP patients between January 2013 and December 2013, including 56 samples collected daily for two weeks from eight patients, with an interval of one day between each sample. Fifty-four were positive for *S. aureus* by quantitative culture (18 MSSA and 36 MRSA specimens). Based on colony counts, 12 MSSA and 28 MRSA isolates were considered pathogenic bacteria, while six MSSA and eight MRSA isolates were involved only in colonization. Ninety-nine samples were negative for *S. aureus* as established by culturing.

Parallel analysis using the multilocus fluorescence-based PCR assay revealed that 69 samples were positive for *S. aureus,* including 19 MSSA specimens with DNA copy numbers ranging from 8.5 *×* 10^2^ to 1.44 *×* 10^7^, and 48 MRSA specimens with DNA copy numbers ranging from 7.20 × 10^3^ to 5.13 × 10^8^. Compared to conventional culturing methods, the real-time PCR assay for MRSA detection demonstrated a sensitivity of 100%, a specificity of 89.6%, a PPV of 75% and NPV of 100% (Table 1).

**Table 1:** Detection of MRSA by bacterial culture and PCR.

	PCR (+)	PCR (−)	Total
MRSA Culture (+)	36	0	36
MRSA Culture (−)	12	106	118
Total	48	106	154

### 3.3 Relationship between Bacterial Levels and PCR Copy Number

We found a positive correlation between MRSA colonies and PCR copy numbers. The average DNA copy number from the 28 MRSA strains identified as HAP pathogens based on PCR analysis was 6.47 × 10^7^. In contrast, the average copy number was only 9.03 × 10^5^ in the remaining eight MRSA samples identified as non-pathogenic colonizers. The average copy number was 1.75 × 10^5^ from 13 MRSA positive cases detected using the quantitative PCR assay. These results reveal a statistical difference in the DNA copy number between the pathogenic and colonizing group. Compared with semi-quantitative culture results, the specificity and sensitivity reached 96.6% and 97.2%, respectively, at the PCR copy number threshold of 5.96 × 10^5^ (Table 2 and Table 3). The detail data about colony counts and DNA copy number for each patient were listed in Supplementary Table S1.

**Table 2:** Correlation between pathogenic or colonizing isolates and DNA copy number.

MRSA Culture	DNA Copy Range				
	0−1 → × 10^4^	1 × 10^4^ to 1 × 10^6^	1 × 10^6^ to 1 × 10^8^	>1 × 10^8^	Average
Infection (n = 28)	0	7 (25%)	18 (64.3%)	3 (10.7%)	6.29 × 10^7^
Colonization (n = 8)	1 (12.5%)	3 (37.5%)	4 (50%)	0	2.31 × 10^6^
PCR (+)/culture (−) (n = 12)	4 (33.3%)	6 (50%)	2 (16.7%)	0	3.10 × 10^5^

**Table 3:** Comparison of sensitivity and specificity of the assay based on different threshold of DNA copies.

DNA Copies	Culture (+)/PCR (+)	Culture (−)/ PCR (−)	Specificity	Sensitivity	PPV	NPV
≥1 × 10^3^	36/48	118/106	89.8%,	100.0%	75%	100%
≥5.96 × 10^5^	35/39	119/115	96.6%	97.2%	89.7%	99.06%

### 3.4 Assessment of Early Diagnosis Based on the Quantitative PCR Assay

Ten patients with HAP were randomly selected, within two weeks of diagnosis, to be subjected to sampling every other day, resulting in seven specimens from each patient. Two cases tested negative for MRSA based on both PCR and bacterial culturing and two tested positive. In the remaining six patients who tested positive for MRSA based on PCR analysis prior to bacterial culturing, the PCR copy number increased from 10^5^ to 10^8^ by days 3–5. In eight patients who were treated with antibiotics targeting MRSA, the positive PCR result remained positive for up to three days after bacterial cultures had become negative (Table 4).

**Table 4:** Evaluation of early diagnosis based on PCR assay results.

Patient No.	Results of PCR (copy number) and Culture (positive/negative)						
	1^st^ detection	2^nd^ detection	3^rd^ detection	4^th^ detection	5^th^ detection	6^th^ detection	7^th^ detection
	(Day 1)	(Day 3)	(Day 5)	(Day 7)	(Day 9)	(Day 11)	(Day 13)
Patient 1	2.09 × 10^6^/N	8.24 × 10^6^/N	2.51 × 10^8^/P	ND	9.06 × 10^7^/P	N/N	N/N
Patient 2	1.71 × 10^6^/N	4.67 × 10^5^/N	9.79 × 10^6^/P	6.00 × 10^4^/P	6.43 × 10^5^/N	N/N	N/N
Patient 3	7.91 × 10^5^/P	5.82 × 10^7^/P	5.88 × 10^4^/N	9.20 × 10^6^/P	1.03 × 10^6^/P	3.06 × 10^5^/N	4.60 × 10^4^/N
Patient 4	7.41 × 10^7^/P	5.07 × 10^6^/P	5.14 × 10^6^/N	3.34 × 10^6^/N	5.14 × 10^6^/N	3.34 × 10^6^/N	N/N
Patient 5	1.57 × 10^5^/N	1.02 × 10^7^/P	9.38 × 10^6^/P	7.45 × 10^6^/P	6.03 × 10^4^/N	1.72 × 10^5^/N	1.65 × 10^5^/N
Patient 6	1.94 × 10^4^/N	1.84 × 10^6^/P	5.63 × 10^5^/N	5.33 × 10^5^/N	8.24 × 10^4^/N	N/N	N/N
Patient 7	3.06 × 10^5^/N	1.01 × 10^7^/P	9.16 × 10^6^/P	1.74 × 10^6^/N	6.62 × 10^5^/N	N/N	N/N
Patient 8	3.76 × 10^6^/N	1.47 × 10^6^/N	1.47 × 10^6^/N	1.88 × 10^7^/P	7.73 × 10^7^/P	4.91 × 10^5^/N	N/N

N indicates a negative culture; P indicates a positive culture positive.

## 4. Discussion

MRSA is one of the most serious pathogens involved in hospital-associated infections. CLSI determined that the presence of MRSA could be confirmed through detection of the *mecA* gene [15]. Detection of *mecA* gene by PCR is regarded as the gold standard for detection of MRSA. A variety of *mecA* gene detection kits have, therefore, been developed for fast detection of MRSA.

The specificity of molecular assays has, however, been subject to debate. Conventional bacterial culturing has been clinically and technically validated for the detection of MRSA. While molecular detection of the *mecA* gene may shorten diagnosis times by approximately one day, the clinical significance of this assay is still questionable, since methicillin resistance-containing strains are so prevalent in the environment and is such common contaminants. It would be beneficial to utilize more rapid and specific molecular assays. Due to the complexity of components and multiple bacterial contaminations leading to high false positive rates, it is challenging to identify the underlying pathogens from clinically isolated specimens, particularly for the diagnosis of HAP. Specimens obtained from the respiratory tract frequently suffer from oral commensal bacterial contamination, leading to a more challenging milieu for molecular assays. For instance, the specificity of GeneXpert is 94.9% and 72.7% for the detection of ETA and MRSA derived from nasal swabs, respectively. Reports for sputum samples are not available [6,16].

Sputum specimens are still the main source for detection of HAP in China even with their limited clinical value. We, therefore, tested a dual gene fluorescence-based PCR assay to directly detect MRSA from sputum samples and ETA. The results showed that the sensitivity and specificity in our study was superior to other reported methods for detection of MRSA. The sensitivity and specificity of the PCR-based method for detection of MRSA and MSSA were 100% accurate in our study. Two specific SCC*mec* genes, *orfX* and *mecA*, are usually used for detection of MRSA in commercial kits, but the former shows false positives in the absence of the *mecA* gene and the latter demonstrates *mecA* positive results in mixtures of MSSA and methicillin-resistant CoNS [17,18].

The *nuc* gene, a target gene detected in our assay, is an alternative target to *S. aureus* SCC*mec* and is not influenced by variation in SCC*mec* or *mecA* gene deletion. Samples that tested positive for the *nuc* gene were subject to subsequent *mecA* detection, thus ensuring that the *nuc* and *mecA* genes were detected from an identical strain, which reduced the risks of false positives as a consequence of mixture with methicillin resistance-containing strains. The type I-V SCC*mec* MRSA reference strain tested positive for *nuc* and *mecA* genes in our study. When mixed with the common bacterial strains residing in the oral cavity and respiratory tract, the control strains of MRconS and streptococci did not yield false positives for *nuc* and *mecA* genes. 

Due to the evolution of MRSA, the SCC*mec* mutation rate has increased resulting in mutation types Vc IX, X and XI. The appearance of *mecA* LGA251 (*mecC*) of SCC*mec* type XI will bring challenges to the molecular diagnosis of MRSA [19,20]. Our fluorescence-based PCR assay was verified to have high specificity and sensitivity in detection of MRSA in clinical specimens. From the 154 specimens isolated from 94 HAP patients, the positive rate was 24% by culture methods and 32.5% by PCR-based assay, which represents no statistically significant difference (*p* > 0.05). Using standard culture methods as the golden standard, the specificity and sensitivity of the PCR-based assay were 89.8% and 100%, respectively, higher than with the use of GeneXpert for detection in ETA from VAP patients (99.0% and 72.2%) [16]. Comparable with other kits, our assay also showed false positives resulting from: (1) contamination of sputum with DNA fragments of the target gene from dead MRSA bacteria; (2) insufficient detection of specific types such as hVISA, which grows slowly [21]; and (3) insufficient detection of MRSA colonies with low copy numbers.

To determine the causes of false positives, we confirmed a positive correlation between the number of bacterial colony forming units and PCR copy numbers. We found that the specimens deemed to be culture positive for bacteria associated with HAP yielded the highest DNA copy numbers, whereas specimens positive only as determined by PCR yielded the lowest DNA copy numbers. Of the 29 HAP bacterial strains, the mean DNA copy number was 6.47 × 10^7^, which was 70- and 370-fold higher than that of the colonized bacterial strains and culture-negative strains, respectively. Our findings were consistent with previous literature reports [16,22]. Further analysis of changes in the DNA copy number of eight patients followed over multiple sampling dates demonstrated similar findings. We conclude that, for culture negative MRSA strains, positive results from the PCR assay indicate potential infection or colonization by MRSA, which warrants continuous monitoring of the changes in DNA copy number. MRSA infection should be suspected particularly based on clinical manifestations. The correlation between DNA copy numbers and bacterial colony forming units, in conjunction with the establishment of the threshold of DNA copy number, may discriminate MRSA infection from colonization.

## 5. Conclusions 

Generally speaking, this is a novel multilocus, fluorescence-based PCR assay, which is proved to be fast, sensitive and specific. Also, it is found to be suitable for routine use in a variety of clinical specimens as well.

## Supplementary Materials

Supplementary materials can be accessed at: http://www.mdpi.com/2076-0817/4/2/199/s1.

## References

[1] Chung D.R., Song J.H., Kim S.H., Thamlikitkul V., Huang S., Wang H., So T.M., Yasin R.M., Hsueh P.R., Carlos C.C. (2011). High prevalence of multidrug-resistant nonfermenters in hospital-acquired pneumonia in Asia. Am. J. Respir. Crit. Care Med..

[2] Zhang X., Wang R., Di X., Liu B., Liu Y. (2014). Different microbiological and clinical aspects of lower respiratory tract infections between China and European/American countries. J. Thorac. Dis..

[3] Engemann J.J., Carmeli Y., Cosgrove S.E., Fowler V.G., Bronstein M.Z., Trivette S.L., Briggs J.P., Sexton D.J., Kaye K.S. (2003). Adverse clinical and economic outcomes attributable to methicillin resistance among patients with Staphylococcus aureus surgical site infection. Clin. Infect. Dis..

[4] Becker K., Pagnier I., Schuhen B., Wenzelburger F., Friedrich A.W., Kipp F., Peters G., von Eiff C. (2006). Does nasal cocolonization by methicillin-resistant coagulase-negative staphylococci and methicillin-susceptible Staphylococcus aureus occur frequently enough to present a risk of false-positive methicillin-resistant Staphylococcus aureus determinations by molecular methods. J. Clin. Microbiol..

[5] Holfelder M., Eigner U., Turnwald A.M., Witte W., Weizenegger M., Fahr A. (2006). Direct detection of methicillin-resistant Staphylococcus aureus in clinical specimens by a nucleic acid-based hybridisation assay. Clin. Microbiol. Infect..

[6] Wolk D.M., Picton E., Johnson D., Davis T., Pancholi P., Ginocchio C.C., Finegold S., Fuller D., Welch D.F., de Boer M. (2009). Multicenter evaluation of the Cepheid Xpert methicillin-resistant Staphylococcus aureus (MRSA) test as a rapid screening method for detection of MRSA in nares. J. Clin. Microbiol..

[7] Patel P.A., Ledeboer N.A., Ginocchio C.C., Condon S., Bouchard S., Qin P., Karchmer T., Peterson L.R. (2011). Performance of the BD GeneOhm MRSA achromopeptidase assay for real-time PCR detection of methicillin-resistant Staphylococcus aureus in nasal specimens. J. Clin. Microbiol..

[8] Katayama Y., Ito T., Hiramatsu K. (2000). A new class of genetic element, staphylococcus cassette chromosome mec, encodes methicillin resistance in Staphylococcus aureus. Antimicrob. Agents Chemother..

[9] Hisata K., Kuwahara-Arai K., Yamanoto M., Ito T., Nakatomi Y., Cui L., Baba T., Terasawa M., Sotozono C., Kinoshita S. (2005). Dissemination of methicillin-resistant staphylococci among healthy Japanese children. J. Clin. Microbiol..

[10] Stürenburg E. (2009). Rapid detection of methicillin-resistant Staphylococcus aureus directly from clinical samples: methods, effectiveness and cost considerations. Ger. Med. Sci..

[11] Wang H., Kim S., Kim J., Park S.-D., Uh Y., Lee H. (2014). Multiplex Real-Time PCR Assay for Rapid Detection of Methicillin- Resistant Staphylococci Directly from Positive Blood Cultures. J. Clin. Microbiol..

[12] Chikkala R., George N.O., Ratnakar K.S., Iyer R.N., Sritharan V. (2012). Heterogeneity in femA in the Indian Isolates of Staphylococcus aureus Limits Its Usefulness as a Species Specific Marker. Adv. Infect. Dis..

[13] American Thoracic Society and the Infectious Diseases Society of America (2005). Guidelines for the management of adults with hospital-acquired, ventilator-associated, and healthcare-associated pneumonia. Am. J. Respir. Crit. Care Med..

[14] Critical care medicine of Chinese Medical Association (2013). Ventilator-associated pneumonia diagnosis, prevention and treatment guidelines (2013). Chin. J. Intern. Med..

[15] Clinical and Laboratory Standards Institute (2012). Performance standards for antimicrobial susceptibility testing: Twenty-second Informational Supplement.

[16] Cercenado E., Marín M., Burillo A., Martín-Rabadán P., Rivera M., Bouza E. (2012). Rapid detection of Staphylococcus aureus in lower respiratory tract secretions from patients with suspected ventilator-associated pneumonia: evaluation of the Cepheid Xpert MRSA/SA SSTI assay. J. Clin. Microbiol..

[17] Blanc D.S., Basset P., Nahimana-Tessemo I., Jaton K., Greub G., Zanetti G. (2011). High proportion of wrongly identified methicillin-resistant Staphylococcus aureus carriers by use of a rapid commercial PCR assay due to presence of staphylococcal cassette chromosome element lacking the mecA gene. J. Clin. Microbiol..

[18] Martineau F., Picard F.J., Roy P.H., Ouellette M., Bergeron M.G. (1998). Species-specific and ubiquitous-DNA-based assays for rapid identification of Staphylococcus aureus. J. Clin. Microbiol..

[19] Li S., Skov R.L., Han X., Larsen A.R., Larsen J., Sørum M., Wulf M., Voss A., Hiramatsu K., Ito T. (2011). Novel types of staphylococcal cassette chromosome mec elements identified in clonal complex 398 methicillin-resistant Staphylococcus aureus strains. Antimicrob. Agents Chemother..

[20] García-Garrote F., Cercenado E., Marín M., Bal M., Trincado P., Corredoira J., Ballesteros C., Pita J., Alonso P., Vindel A. (2014). Methicillin-resistant Staphylococcus aureus carrying the mecC gene: Emergence in Spain and report of a fatal case of bacteraemia. J. Antimicrob. Chemother..

[21] Wang Y., Hu Y.J., Ai X.M., Xu H.T., Sun T.Y. (2013). Prevalence and clinical prognosis of heteroresistant vancomycin-intermediate Staphylococcus aureus in a tertiary care center in China. Chin. Med. J. Engl..

[22] McConnell M.J., Pérez-Ordóñez A., Pérez-Romero P., Valencia R., Lepe J.A., Vázquez-Barba I., Pachón J. (2014). Quantitative real-time PCR for detection of Acinetobacter baumannii colonization in the hospital environment. J. Clin. Microbiol..

