# Supplementary material for: Direct Detection of Methicillin-Resistant Staphylococcus Aureus in Sputum Specimens from Patients with Hospital-Associated Pneumonia Using a Novel Multilocus Pcr Assay
Source: Pathogens. 2015 Apr 30;4(2):199–209. doi: 10.3390/pathogens4020199 (PMC4493470; doi:10.3390/pathogens4020199)
Supplement: Supplementary file 1 [file pathogens-04-00199-s001.pdf]

Supplementary Materials

Table S1. Correlation between colony counts and DNA copy number.

No.	MRSA culture	Sample type	DNA Copy	Colony counts(CFU/mL)
1	Infection	SP	7.73×10^{7}	7.26×10^{7}
2	Infection	SP	7.62×10^{7}	5.19×10^{7}
3	Infection	ETA	1.84×10^{6}	2.68×10^{6}
4	Infection	SP	5.82×10^{7}	6.46×10^{7}
5	Infection	ETA	7.91×10^{5}	2.51×10^{5}
6	Infection	SP	5.23×10^{8}	6.11×10^{8}
7	Infection	SP	1.02×10^{7}	1.56×10^{7}
8	Infection	ETA	9.01×10^{6}	2.86×10^{6}
9	Infection	SP	7.41×10^{7}	7.01×10^{7}
10	Infection	ETA	4.80×10^{5}	1.46×10^{5}
11	Infection	SP	9.06×10^{7}	1.06×10^{8}
12	Infection	ETA	8.65×10^{6}	5.56×10^{6}
13	Infection	SP	1.01×10^{7}	9.89×10^{6}
14	Infection	SP	2.67×10^{8}	1.13×10^{8}
15	Infection	SP	8.57×10^{7}	8.89×10^{7}
16	Infection	ETA	9.79×10^{6}	1.45×10^{7}
17	Infection	SP	9.20×10^{6}	3.12×10^{6}
18	Infection	SP	1.03×10^{6}	8.05×10^{5}
19	Infection	ETA	1.88×10^{7}	1.03×10^{7}
20	Infection	SP	8.03×10^{6}	2.15×10^{6}
21	Infection	SP	2.51×10^{8}	2.11×10^{8}
22	Infection	ETA	7.10×10^{4}	5.84×10^{4}
23	Infection	SP	5.07×10^{6}	2.56×10^{6}
24	Infection	SP	9.06×10^{7}	6.57×10^{7}
25	Infection	ETA	1.27×10^{5}	9.09×10^{4}
26	Infection	SP	8.71×10^{5}	3.65×10^{5}
27	Infection	SP	5.52×10^{5}	2.98×10^{5}
28	Infection	ETA	6.67×10^{4}	3.82×10^{4}
29	Colonization	SP	3.77×10^{4}	1.79×10^{4}
30	Colonization	SP	3.74×10^{6}	2.98×10^{6}
31	Colonization	SP	5.94×10^{6}	3.71×10^{6}
32	Colonization	SP	9.63×10^{5}	8.45×10^{5}
33	Colonization	SP	3.49×10^{6}	1.96×10^{6}
34	Colonization	SP	4.08×10^{6}	2.65×10^{6}
35	Colonization	ETA	2.27×10^{4}	2.08×10^{4}
36	Colonization	ETA	7.20×10^{3}	6.57×10^{3}

Table S1. *Cont.*

No.	MRSA culture	Sample type	DNA Copy	Colony counts(CFU/mL)
37	PCR (+)/culture (−)	SP	2.01×10^4	1.95×10^4
38	PCR (+)/culture (−)	SP	2.72×10^4	2.09×10^4
39	PCR (+)/culture (−)	SP	1.02×10^6	8.65×10^5
40	PCR (+)/culture (−)	SP	1.95×10^4	1.09×10^4
41	PCR (+)/culture (−)	SP	9.37×10^3	6.75×10^3
42	PCR (+)/culture (−)	SP	1.09×10^5	9.76×10^4
43	PCR (+)/culture (−)	SP	1.50×10^3	1.42×10^3
44	PCR (+)/culture (−)	SP	8.75×10^2	6.89×10^2
45	PCR (+)/culture (−)	SP	4.16×10^3	3.28×10^3
46	PCR (+)/culture (−)	SP	3.17×10^4	3.92×10^4
47	PCR (+)/culture (−)	SP	2.51×10^6	1.86×10^6
48	PCR (+)/culture (−)	SP	1.87×10^4	1.16×10^4
